# 30-Day mortality risk predictors for emergency laparotomy: a comparative study

**DOI:** 10.1308/rcsann.2025.0075

**Published:** 2026-01-12

**Authors:** M Hassan, K AbdelSaid, AK Ebrahim, B Jayasankar, M Riad, M Jeilani, Y Abdul Aal

**Affiliations:** Maidstone and Tunbridge Wells NHS Trust, UK

**Keywords:** Laparotomy, ACS-NSQIP, SORT, P-POSSUM, NELA

## Abstract

**Introduction:**

Morbidity and mortality are significant risks associated with emergency laparotomies. A risk calculation tool facilitates the identification of high-risk patients and provides clinicians with information to help them make informed decisions. In search of an ideal scoring system that yields accurate results, we compared 30-day mortality predictions using the National Emergency Laparotomy Audit (NELA), the Physiological and Operative Severity Score for the Enumeration of Mortality and Morbidity (P-POSSUM), the American College of Surgeons National Surgical Quality Improvement Program (ACS-NSQIP), and the Surgical Outcome Risk Tool (SORT) risk calculators.

**Methods:**

This retrospective study analysed data collected from adult patients who underwent emergency laparotomies between July 2018 to October 2019 at Maidstone and Tunbridge Wells NHS Trust. Each patient’s median preoperative mortality risk was calculated using the four risk calculators: NELA, P-POSSUM, ACS-NSQIP and SORT.

**Results:**

During the study period, 227 patients were eligible for inclusion, with a mean (sd) age of 65 (±16) years and a median American Society of Anesthesiologists score of 2. NELA and P-POSSUM identified 11 patients (sensitivity 73.3%) who died in the high-risk group, which was higher than the identification rates of ACS-NSQIP (53.3%) and SORT (40.0%). The average 30-day mortality risk for the 15 patients who died was 25.8% for NELA, 39.6% for P-POSSUM, 17.9% for ACS-NSQIP and 15.7% for SORT. NELA and ACS-NSQIP had the highest area under the curve at 0.869 and 0.877, respectively. Although NELA exhibited higher sensitivity (73.3%), ACS-NSQIP demonstrated greater specificity (88.7%).

**Conclusions:**

Overall, the NELA score demonstrated the highest performance in predicting mortality in emergency laparotomy.

## Introduction

Emergency laparotomy is associated with significant morbidity and mortality, and the mortality rate among patients requiring emergency laparotomies remains high despite advances in medicine and surgery. Numerous national and international efforts have been undertaken to reduce the mortality and morbidity associated with these procedures. As part of these initiatives, audits and quality improvement programmes have been conducted within and across hospital centres to develop an optimal scoring system that accurately estimates mortality risk. Although many scoring systems have been developed, no single system has been validated across multiple centres or geographical locations. Some scoring systems predict risk based solely on preoperative findings and parameters, whereas others rely on intraoperative assessments and histopathological reports for accurate mortality risk stratification. The National Emergency Laparotomy Audit (NELA) was established using data from England and Wales, and has become a standard for comparison in other population groups. These data led to the development of the NELA Risk Prediction Calculator (NRPC) to assess 30-day mortality and morbidity risk.^1–3^ Based on these data, the NRPC has been developed to estimate 30-day mortality and morbidity risks and is one of several risk prediction tools available.^4^ Other commonly used risk calculators include the Portsmouth Physiological and Operative Severity Score for the Enumeration of Mortality and Morbidity (P-POSSUM), the American College of Surgeons National Surgical Quality Improvement Program (ACS-NSQIP) and the Surgical Outcome Risk Tool (SORT).^4–6^ An ideal scoring system would allow for accurate outcome predictions, enabling the treating team to present informed choices to patients regarding surgery or supportive care.^7^ In addition, it should facilitate risk-adjusted comparisons among surgeons, hospitals and geographical distributions.^8^ However, there is no consensus on which scoring system best predicts mortality risk in emergency laparotomies.^9^ This study aims to evaluate the performance of four widely used mortality risk prediction tools, NELA, P-POSSUM, ACS-NSQIP and SORT, in predicting 30-day mortality among patients undergoing emergency laparotomy at Maidstone and Tunbridge Wells NHS Trust.

## Methods

### Study design and setting

We conducted a retrospective analysis of all emergency laparotomies performed at our trust, a typical district general hospital in the UK. The study included all consecutive emergency laparotomies performed on adults aged 18 or older from July 2018 to October 2019.

### Inclusion and exclusion criteria

We applied the NELA inclusion and exclusion criteria for patient selection, focusing on major emergency abdominal general surgery procedures performed on adult patients.^10^

### Data collection

To estimate the 30-day mortality risk percentages, preoperative data were entered into the online NRPC, ACS-NSQIP, P-POSSUM and SORT calculators (see Appendix 1, available online, for a list of online calculators and parameters). Collected data included patient demographics, presentation, preoperative investigations, procedural details and outcomes (Table 1). Patients without adequate preoperative investigations were excluded from the study.

**Table 1:** Demographic data of the studied patients (*N* = 227)

Variable	
Age, mean (sd)	65.0 (16.2)
Gender, *n* (%)	
Female	126 (63.5)
Male	101 (36.5)
ASA, mean (sd)	2.41 (0.93)

ASA = American Society of Anesthesiologists; sd = standard deviation

### Statistical analyses

Descriptive statistics were used to summarise the demographic and clinical characteristics of the study population. Continuous variables were compared using Student’s *t*-test, and categorical variables were compared using the chi-squared test. The performance of each mortality risk prediction tool was evaluated using receiver operating characteristic curves, sensitivity, specificity and area under the curve (AUC). Statistical significance was set at *p* < 0.05. Statistical analyses were performed using SPSS version 25.0.

### Outcome

The outcome of the study was to calculate the discrimination of each of the selected risk prediction tools. Any death occurring within 30 days of surgery was considered postoperative mortality.

## Results

A total of 227 patient charts were included in the study period. Patient demographics included 101 men (36.5%), with a mean (sd) age of 65 (±16) years and a median American Society of Anesthesiologists (ASA) score of 2 (interquartile range [IQR] 2–3), indicating that the majority of patients had moderate systemic disease, and a mean (sd) length of stay of 17.48 (±16.23) days (Table 1).

The distribution of surgical procedures is shown in Table 2, with small bowel resections (19.8%) and adhesiolysis (17.6%) being the most common surgery.

**Table 2:** List of most common operations

Operation	No. performed (%)
Small bowel resection	45 (19.8)
Adhesiolysis	40 (17.6)
Hartmann’s procedure	37 (16.3)
Right hemicolectomy	34 (15)
Colorectal resection – other/not specified	20 (8.8)
Defunctioning stoma	9 (4.0)
Repair of peptic ulcer	8 (3.5)
Exploratory/relook laparotomy	8 (3.5)
Repair of intestinal perforation	6 (2.6)
Incisional hernia repair	6 (2.6)
Other procedures (<5 cases of each)	14 (16.7)
Total procedures	227 (100)

Of the 227 patients, 15 (6.6%) died within 30 days post-surgery. Patients who died had a significantly higher mean age of 72.93 years compared with 64.43 years for survivors (*p* < 0.05). Similarly, the mean ASA score was higher in those who died (3.33) than in survivors (2.34) (*p* < 0.05; Table 3).

**Table 3:** Relation of mortality with demographic data and characteristics of the studied patients

Variables	Mortality		*p*-value	Significance
	Yes	No		
	*N* = 15	*N* = 212		
Age, mean (sd)			0.049^†^	S
Gender, *n* (%)	72.93 (14.70)	64.43 (16.22)		
Female	5 (33.3%)	121 (57.1%)	0.015^*^	S
Male	10 (66.7%)	91 (42.9%)		
ASA, mean (sd)	3.33 (1.11)	2.34 (0.88)	0.000^†^	HS

ASA = American Society of Anesthesiologists; HS = highly significant (*p* < 0.01); S = significant (*p* < 0.05); sd = standard deviation

*Chi-squared test

^†^Independent *t*-test

**Figure 1:**
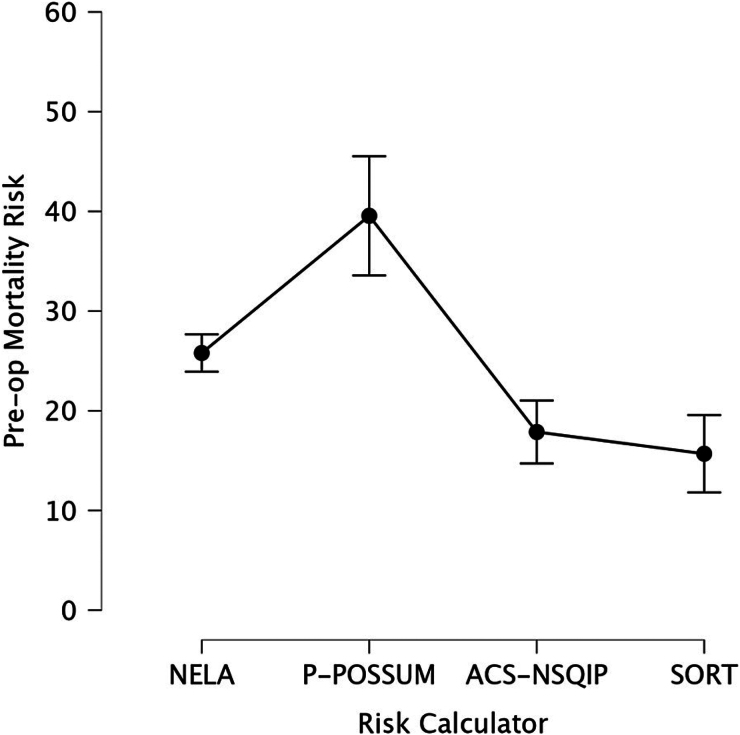
Plot of average preoperative mortality risk of those who died (%), with confidence intervals. ACS-NSQIP = the American College of Surgeons National Surgical Quality Improvement Program; NELA = National Emergency Laparotomy Audit; P-POSSUM = Portsmouth Physiological and Operative Severity Score for the Enumeration of Mortality and Morbidity; SORT = The Surgical Outcome Risk Tool

The effectiveness of each mortality risk calculator in predicting 30-day mortality is summarised in Table 4. Both NELA and P-POSSUM identified 73.3% of the deaths as high risk, whereas ACS-NSQIP and SORT identified 53.3% and 40.0% of the deaths as high risk, respectively (Table 4). The mean predicted 30-day mortality risk for patients who died was highest for P-POSSUM (39.6%), followed by NELA (25.8%), ACS-NSQIP (17.9%) and SORT (15.7%) (Figure 1). All four scores demonstrated significant differences in mortality between high- and low-risk groups using the 10% cut-off mark.

**Table 4:** Relation of mortality with preoperative mortality risk scores and stratification of the studied patients

Risk prediction tool	Mortality		*p*-value	Significance
	Yes	No		
	*N* = 15	*N* = 212		
NELA				
Mean (sd)	25.79 (22.40)	9.93 (6.54)	0.000^†^	HS
High, *n* (%)	11 (73.3)	39 (18.4)	0.000^*^	HS
Low, *n* (%)	4 (26.7)	173 (81.6)		
Mean (sd)	39.55 (35.52)	11.81 (17.09)	0.000^†^	HS
P-POSSUM				
High, *n* (%)	11 (73.3)	63 (29.7)	0.000^*^	HS
Low, *n* (%)	4 (26.7)	149 (70.3)		
Mean (sd)	17.87 (16.76)	5.26 (4.00)	0.000^†^	HS
ACS-NSQIP				
High, *n* (%)	8 (53.3)	24 (11.3)	0.000^*^	HS
Low, *n* (%)	7 (46.7)	188 (88.7)		
Mean (sd)	15.69 (15.98)	7.28 (5.14)	0.000^†^	HS
SORT				
High, *n* (%)	6 (40.0)	38 (17.9)	0.000^*^	HS
Low, *n* (%)	9 (60.0)	174 (82.1)		

ACS-NSQIP = American College of Surgeons National Surgical Quality Improvement Program; HS = highly significant (*p* < 0.01); NELA = National Emergency Laparotomy Audit; P-POSSUM = Portsmouth Physiological and Operative Severity Score for the Enumeration of Mortality and Morbidity; SORT = Surgical Outcome Risk Tool

*Chi-squared test

^†^Independent *t*-test

High ≥10%; low <10%

**Figure 2:**
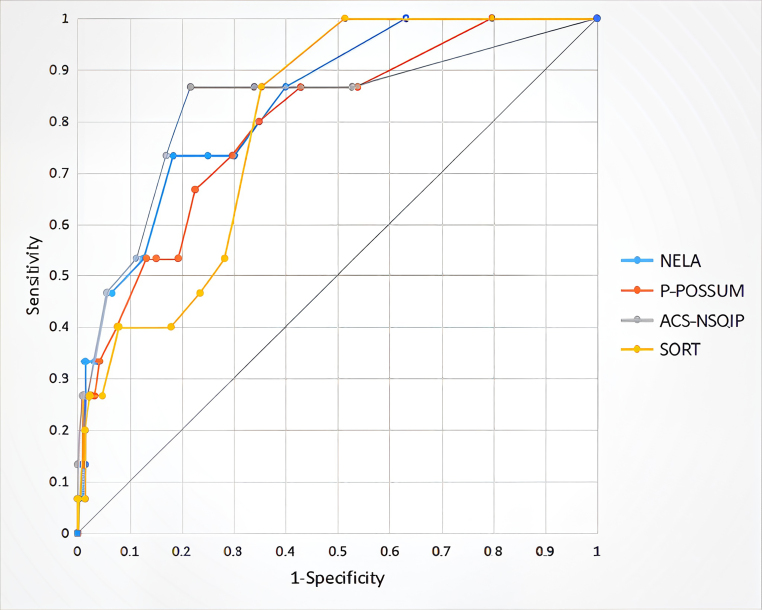
Receiver operating characteristic curve for the four mortality scores in differentiating between 30-day mortality and survival. ACS-NSQIP = the American College of Surgeons National Surgical Quality Improvement Program; NELA = National Emergency Laparotomy Audit; P-POSSUM  = Portsmouth Physiological and Operative Severity Score for the Enumeration of Mortality and Morbidity; SORT = Surgical Outcome Risk Tool

P-POSSUM vs ACS-NSQIP and P-POSSUM vs SORT showed statistically significant differences in 30-day mortality risk (Table 5). NELA and ACS-NSQIP had the highest AUC at 0.869 and 0.877, respectively. NELA exhibited higher sensitivity (73.3%), while ACS-NSQIP demonstrated greater specificity (88.7%). Optimal cut-off scores were calculated using Youden’s index (Figure 2 and Table 6).

**Table 5:** Comparison between the preoperative mortality risk scores of those who died

	95% CI for mean difference					
	Mean Difference	Lower	Upper	SE	t	*p*_Bonf_
NELA						
P-POSSUM	−13.760	−29.455	1.935	5.668	−2.428	0.117
ACS-NSQIP	7.920	−7.775	23.615	5.668	1.397	1.000
SORT	10.103	−5.593	25.798	5.668	1.782	0.492
P-POSSUM						
ACS-NSQIP	21.680	5.985	37.375	5.668	3.825	0.003
SORT	23.863	8.167	39.558	5.668	4.210	
ACS-NSQIP						
SORT	2.183	−13.513	17.878	5.668	0.385	1.000

The *p*-value and confidence intervals (CI) are adjusted for comparing a family of six estimates (CI corrected using the Bonferroni method)

ACS-NSQIP = American College of Surgeons National Surgical Quality Improvement Program; NELA = National Emergency Laparotomy Audit; P-POSSUM = Portsmouth Physiological and Operative Severity Score for the Enumeration of Mortality and Morbidity; SORT = The Surgical Outcome Risk Tool

**Table 6:** Comparison of the four mortality scores in predicting 30-day mortality

Risk prediction tool	Cut-off point	AUC	Sensitivity (%)	Specificity (%)	PPV (%)	NPV (%)
NELA	>8%	0.869	73.3	81.6	22.0	97.7
P-POSSUM	>6%	0.828	73.3	70.3	14.9	97.4
ACS-NSQIP	>4%	0.877	53.3	88.7	25.0	96.4
SORT	>2%	0.822	40.0	82.1	13.6	95.1

ACS-NSQIP = the American College of Surgeons National Surgical Quality Improvement Program; AUC = area under the curve; NELA = National Emergency Laparotomy Audit; NPV = negative predictive value; P-POSSUM = Portsmouth Physiological and Operative Severity Score for the Enumeration of Mortality and Morbidity; PPV = positive predictive value; SORT = Surgical Outcome Risk Tool

## Discussion

Emergency laparotomy is one of the most critical surgical interventions performed in the acute setting. Despite its frequency, it carries a disproportionately high morbidity and mortality burden compared with elective general surgery procedures. Accurate risk prediction tools are thus essential for guiding clinical decision making, optimising perioperative planning and improving patient outcomes.

A variety of scoring systems have been developed to estimate mortality risk in emergency laparotomy. Among these, the NELA, P-POSSUM, SORT and ACS-NSQIP calculator are the most commonly used in clinical practice. This study compared these four tools in a UK district general hospital cohort and assessed their predictive accuracy for 30-day mortality.

Our results demonstrate that NELA exhibited the highest AUC (0.869), indicating strong discriminative ability for predicting 30-day mortality. This aligns with findings from Thahir *et al* and Odor and Grocott, who reported superior performance of NELA in emergency general surgery settings.^9,11^ In a Singaporean cohort, Lai *et al* found that whereas both NELA and P-POSSUM over-predicted mortality, NELA more closely mirrored observed outcomes.^12^

P-POSSUM, long considered a robust scoring tool, showed slightly lower predictive accuracy (AUC = 0.828) in our study. This is consistent with previous literature acknowledging its general reliability but also its tendency to overestimate mortality – particularly in lower-risk or elderly populations.^13–16^ Studies by Özban *et al* and Stonelake *et al* have similarly identified this limitation.^17,18^ However, Barazanchi *et al* demonstrated that combining P-POSSUM with frailty and nutritional assessments improved its predictive value, especially in non-UK populations.^19^

SORT and ACS-NSQIP showed inferior discriminative power in our cohort, with ACS-NSQIP having the highest specificity. This may make it suitable for stratifying low-risk patients, as noted by Eliezer *et al* in their Australian study, in which ACS-NSQIP performed well in lower-risk patients but lacked sensitivity for identifying those at high risk of early mortality.^20^

Age and ASA score emerged as significant predictors of 30-day mortality in our study. These findings are in line with global studies identifying age, physiological reserve and comorbidity burden as key mortality determinants in emergency surgical patients.^1,2^ In particular, Moscalu *et al* highlighted that NELA is more accurate in patients younger than 70 years, suggesting reduced utility in elderly populations, a trend mirrored by our data.^21^

Frailty scoring is increasingly recognised as a valuable adjunct to traditional risk models. Isand *et al* showed that the Clinical Frailty Scale, when combined with NELA or P-POSSUM, substantially improved risk stratification in older patients.^22^ The incorporation of such metrics into mainstream calculators may represent the next evolution in risk modelling for emergency laparotomy.

Our findings support the use of NELA as the most effective predictor of 30-day mortality following emergency laparotomy in UK clinical practice. However, the choice of scoring system should be context specific. P-POSSUM and ACS-NSQIP may retain value when supplemented by clinical insight and additional patient data such as frailty and nutritional status. Importantly, no single tool should replace surgical judgement, especially in resource-variable environments.

### Study limitations

The retrospective design and single-centre scope of this study may limit the generalisability of our findings. The relatively small sample size and the inherent biases associated with retrospective data collection also pose limitations. Future research involving multicentre studies with larger patient cohorts is necessary to validate these findings and develop more universally applicable risk prediction models.

## Conclusions

In conclusion, although NELA demonstrates the highest overall accuracy for predicting 30-day mortality in emergency laparotomy patients, no single risk calculator is definitively superior. Each tool has its strengths and limitations, necessitating the use of clinical judgement in conjunction with risk predictions for optimal patient management. Future research should aim to validate these findings in larger, multicentre cohorts and explore the integration of advanced predictive algorithms to further improve risk stratification.

## Conflict of interest

All authors declare that there is no conflict associated with this publication and that they did not receive any significant funding that would influence the study.
